# Association of *Helicobacter pylori* Infection and Risk of Dyslipidemia: A Systematic Review and Meta‐Analysis

**DOI:** 10.1002/jgh3.70128

**Published:** 2025-03-24

**Authors:** Ankita Gaonkar, Quazi Syed Zahiruddin, Muhammed Shabil, Soumya V. Menon, Mandeep Kaur, Mukesh Kumari, Puneet Sudan, K. Satyam Naidu, Shailendra Thapliyal, Jyoti Uikey, Rachna Kathuria, Sanjay Singh Chauhan, Lokesh Verma, Amritpal Sidhu, Ganesh Bushi, Rohimah Binti Md Yusoff, Rachana Mehta, Prakasini Satapathy, Sanjit Sah

**Affiliations:** ^1^ Noida Institute of Engineering and Technology (Pharmacy Institute) Greater Noida India; ^2^ South Asia Infant Feeding Research Network (SAIFRN), Division of Evidence Synthesis, Global Consortium of Public Health and Research Datta Meghe Institute of Higher Education Wardha India; ^3^ University Center for Research and Development, Chandigarh University Mohali Punjab India; ^4^ Medical Laboratories Techniques Department, AL‐Mustaqbal University Babil Iraq; ^5^ Department of Chemistry and Biochemistry School of Sciences JAIN (Deemed to be University) Bangalore Karnataka India; ^6^ Department of Allied Healthcare and Sciences Vivekananda Global University Jaipur Rajasthan India; ^7^ Department of Applied Sciences‐Chemistry NIMS Institute of Engineering & Technology, NIMS University Rajasthan Jaipur India; ^8^ Department of Pharmacy Chandigarh Pharmacy College Chandigarh Group of Colleges‐Jhanjeri Mohali Punjab India; ^9^ Department of Chemistry Raghu Engineering College Visakhapatnam Andhra Pradesh India; ^10^ Uttaranchal Institute of Management Uttaranchal University Dehradun Uttarakhand India; ^11^ IES Institute of Pharmacy IES University Bhopal Madhya Pradesh India; ^12^ New Delhi Institute of Management, Tughlakabad Institutional Area New Delhi India; ^13^ Department of Microbiology Graphic Era (Deemed to Be University) Dehradun India; ^14^ Centre of Research Impact and Outcome Chitkara University Rajpura Punjab India; ^15^ Chitkara Centre for Research and Development Chitkara University Rajpura Himachal Pradesh India; ^16^ School of Pharmaceutical Sciences Lovely Professional University Phagwara India; ^17^ University of Cyberjaya Persiaran Bestari, Cyberjaya Selangor Darul Ehsan Malaysia; ^18^ Clinical Microbiology RDC, Manav Rachna International Institute of Research and Studies Faridabad Haryana India; ^19^ Center for Global Health Research, Saveetha Medical College and Hospital, Saveetha Institute of Medical and Technical Sciences Saveetha University Chennai India; ^20^ University of Cyberjaya, Persiaran Bestari Cyberjaya Selangor Darul Ehsan Malaysia; ^21^ SR Sanjeevani Hospital Kalyanpur Nepal; ^22^ Department of Paediatrics Dr. D. Y. Patil Medical College, Hospital and Research Centre, Dr. D. Y. Patil Vidyapeeth Pune Maharashtra India; ^23^ Department of Public Health Dentistry Dr. D.Y. Patil Dental College and Hospital, Dr. D.Y. Patil Vidyapeeth Pune Maharashtra India

**Keywords:** dyslipidemia, HDL‐C, *Helicobacter pylori*, LDL‐C, lipids, total cholesterol

## Abstract

**Background:**

Although *Helicobacter pylori* ( *H. pylori* ) infections are widespread throughout the world, it is yet unknown whether they are linked to systemic illnesses like dyslipidemia. The purpose of this systematic review and meta‐analysis was to examine the connection between lipid metabolism and *H. pylori* infection, with a particular emphasis on how it affects dyslipidemia.

**Methods:**

We conducted a thorough search up until October 10, 2024, across databases such as PubMed, Web of Science, and Embase. Studies that reported lipid profiles in both *H. pylori* ‐infected and non‐infected patients were considered eligible. The primary outcomes were triglyceride, LDL‐C, HDL‐C, and total cholesterol levels, which were examined using a random‐effects model in R software version 4.4.

**Results:**

There were 17 studies with more than 150,000 participants from 681 screened publications. Higher levels of LDL (MD: 5.32 mg/dL; 95% CI: 1.315 to 9.319) and total cholesterol (MD: 6.28 mg/dL; 95% CI: 0.718 to 11.842), as well as lower levels of HDL (MD: −2.06 mg/dL; 95% CI: −3.212 to −0.915), were the results of the meta‐analysis. Among those infected, triglyceride levels were likewise higher (MD: 7.93 mg/dL; 95% CI: 0.413 to 15.436), but the odds ratio (OR) did not show a significant increase in risk (OR: 1.002; 95% CI: 0.995 to 1.010).

**Conclusion:**

*H. pylori* infection is associated with significant dyslipidemia, suggesting a potential link between chronic bacterial infection and lipid metabolism. The findings emphasize the need for further research to explore the mechanisms and potential therapeutic interventions.

## 1 Introduction

*H. pylori* is known for its high prevalence across different regions, particularly in developing countries where up to 80% of adults may be infected [1]. The transmission of *H. pylori* primarily occurs through oral –oral or fecal –oral routes, often facilitated by poor sanitation, overcrowding, and inadequate healthcare resources [2]. Despite its widespread presence, many individuals remain asymptomatic, and the bacterium can persist in the stomach for decades without causing overt clinical symptoms [3]. *H. pylori* 's pathogenic potential is highlighted by its capacity to adapt to the harsh, acidic environment of the stomach and its defense mechanisms against the host immune system [4]. But in addition to being linked to local stomach disorders, *H. pylori* also seems to have an impact on overall health [5]. The mechanisms through which *H. pylori* induces gastric pathology are well documented and include chronic inflammation, mucosal barrier disruption, and the production of virulence factors like CagA and VacA [6]. According to recent research, *H. pylori* may be involved in extra‐gastrointestinal disorders such as metabolic syndrome, idiopathic thrombocytopenic purpura, and iron deficiency anemia [7]. Specifically, an increasing amount of evidence indicates that dyslipidemia may result from changes in lipid metabolism brought on by an *H. pylori* infection [8]. Although the precise processes by which *H. pylori* may affect lipid levels are not entirely understood, they may include insulin resistance, chronic low‐grade inflammation, and the impact of bacterial toxins on lipid synthesis and metabolism [9].

Dyslipidemia is characterized by elevated levels of low‐density lipoprotein cholesterol (LDL‐C), triglycerides (TG), total cholesterol, or High‐density lipoprotein cholesterol (HDL‐C) [10]. These lipid abnormalities are well‐known risk factors for cardiovascular diseases, which account for a significant proportion of global mortality [11]. According to the World Health Organization (WHO), cardiovascular illnesses cause around 17.9 million deaths a year, or 31% of all fatalities globally [12]. The growing burden of dyslipidemia, driven by lifestyle factors such as poor diet, lack of physical activity, and increasing prevalence of obesity, underscores the need for a better understanding of its risk factors, including potential infectious contributors like *H. pylori* [13]. The hypothesis that *H. pylori* infection may be linked to dyslipidemia stems from its ability to induce a state of chronic inflammation, which has been associated with metabolic disturbances [14]. Chronic inflammatory states can interfere with normal lipid metabolism, promoting changes such as increased triglycerides and LDL‐C, alongside decreased HDL‐C [15]. While some research has identified no significant correlation, other studies argue that *H. pylori* infection is associated with higher LDL‐C and lower HDL‐C levels [16]. The exact strain of *H. pylori* implicated, variations in study design, population characteristics, and other confounding variables like age, food, and lifestyle could all be to blame for this discrepancy [17].

Moreover, it has been proposed that the presence of specific *H. pylori* virulence factors, particularly CagA, may play a role in the metabolic disturbances observed in infected individuals [18]. CagA‐positive strains have been shown to induce more severe inflammatory responses, which might exacerbate alterations in lipid metabolism [19]. Furthermore, *H. pylori* infection has been linked to alterations in gut microbiota, which are known to affect metabolic processes, including lipid metabolism [9]. Therefore, the possible connection between *H. pylori* and dyslipidemia could be complex, comprising a complex interplay between environmental, host, and bacterial components [20].

Given the high global prevalence of both *H. pylori* infection and dyslipidemia, understanding whether a significant association exists between the two could have important implications for public health [21]. If a link is established, it may prompt further investigation into the mechanisms involved and potentially open new avenues for the prevention and management of dyslipidemia through the treatment of *H. pylori* infection [22]. In order to synthesize the available information, determine the strength of the link, and identify knowledge gaps that warrant further investigation, a systematic review and meta‐analysis of recent studies would be beneficial [23]. By examining data from earlier research, this systematic review and meta‐analysis sought to determine the association between *H. pylori* infection and the risk of dyslipidemia.

## 2 Methods

For reporting this systematic review, we followed the Preferred Reporting Items for Systematic Reviews and Meta‐Analyses (PRISMA) standards (Table S1) [24]. In PROSPERO, a protocol has been prospectively registered: CRD42024586379.

### 2.1 Search Strategy

A comprehensive literature search was performed to identify studies relevant to the association between *H. pylori* infection and dyslipidemia. The search was conducted across various electronic databases, including PubMed, Embase, and Web of Science, up to October 10, 2024. Keywords and Medical Subject Headings (MeSH) terms such as “ *Helicobacter pylori* ,” “dyslipidemia,” “hyperlipidemia,” “cholesterol,” “lipid profile,” and their variations were used to ensure a broad search. No restrictions were applied to the publication date or language of articles. The complete search strategy used for each database is presented in Table S2.

### 2.2 Inclusion and Exclusion Criteria

Studies were included in the analysis if they met specific criteria. First, it was necessary to examine the relationship between *H. pylori* infection and dyslipidemia or to report alterations in lipid profiles. Second, only studies that presented original data from observational designs, such as cross‐sectional, case–control, or cohort studies, were considered. Third, the studies needed to provide adequate data to calculate odds ratios (ORs) or mean differences (MDs), along with the corresponding 95% confidence intervals (CIs). Conversely, review articles, editorials, or case reports were excluded if they lacked control groups or failed to provide adequate data for meta‐analysis.

### 2.3 Screening

Initially, all records identified through the database searches were imported into a semi‐automated web software (Nested‐Knowledge) to facilitate organization, removal of duplicates, and screening. Two independent reviewers reviewed the titles and abstracts of all studies retrieved to evaluate their relevance in line with the established inclusion and exclusion criteria. During this initial screening phase, studies were quickly evaluated to determine whether they potentially addressed the association between *H. pylori* infection and dyslipidemia. Articles that clearly did not meet the inclusion criteria, such as those focused on unrelated health conditions, non‐human studies, or those not reporting original research data, were excluded at this stage. If there was any uncertainty regarding the relevance of a study, it was retained for further evaluation during the next phase of screening. For the studies that passed the initial title and abstract review, the full‐text articles were obtained for a more detailed assessment. The same two reviewers independently reviewed the full‐text versions to ensure a comprehensive evaluation of each study's methodology, population characteristics, and outcome measures. During this phase, the reviewers checked for essential criteria, including whether the study specifically examined the relationship between *H. pylori* infection and lipid abnormalities, employed a suitable observational study design (cross‐sectional, case–control, or cohort), and provided sufficient statistical data to allow for the calculation of effect measures like ORs or MDs with 95% CIs. To minimize selection bias and ensure consistency, any differing opinions between the two reviewers regarding study eligibility were addressed through discussion. If they were unable to reach a consensus, another reviewer was brought in to make the final decision.

### 2.4 Data Extraction and Quality Assessment

Two individual reviewers (AG, MS) independently examined the titles and abstracts of the candidate studies to evaluate their eligibility. Full‐text articles of studies deemed potentially relevant were then retrieved and carefully examined to confirm inclusion. Data extraction was completed using a standardized form, collecting details on study details (author, year, country, sample size, study design) and participant demographics (age, gender) diagnostic methods for *H. pylori* infection, and lipid profile outcomes (total cholesterol, triglycerides, HDL‐C, and LDL‐C). Disagreements between the reviewers were addressed through discussion or, if required, by seeking the opinion of another reviewer (QSZ). The Newcastle‐Ottawa Scale (NOS) was used to assess the quality of the included studies. This scale evaluates observational studies based on criteria such as the selection of study groups, the comparability of groups, and the assessment of outcomes.

### 2.5 Statistical Analysis

Meta‐analysis was performed using R software version 4.4 [25]. Studies reporting ORs with 95% CIs were pooled to examine the association between *H. pylori* infection and dyslipidemia, while MDs with 95% CIs were used to compare lipid levels between *H. pylori* ‐infected and non‐infected individuals for continuous outcomes. Heterogeneity between studies was evaluated using the I^2^ statistic. Values of 25%, 50%, and 75% for I^2^ indicate low, moderate, and high levels of heterogeneity, respectively. A random‐effects model was used for the meta‐analysis, and publication bias was assessed using funnel plots and Egger's test.

## 3 Results

### 3.1 Literature Search

**FIGURE 1:**
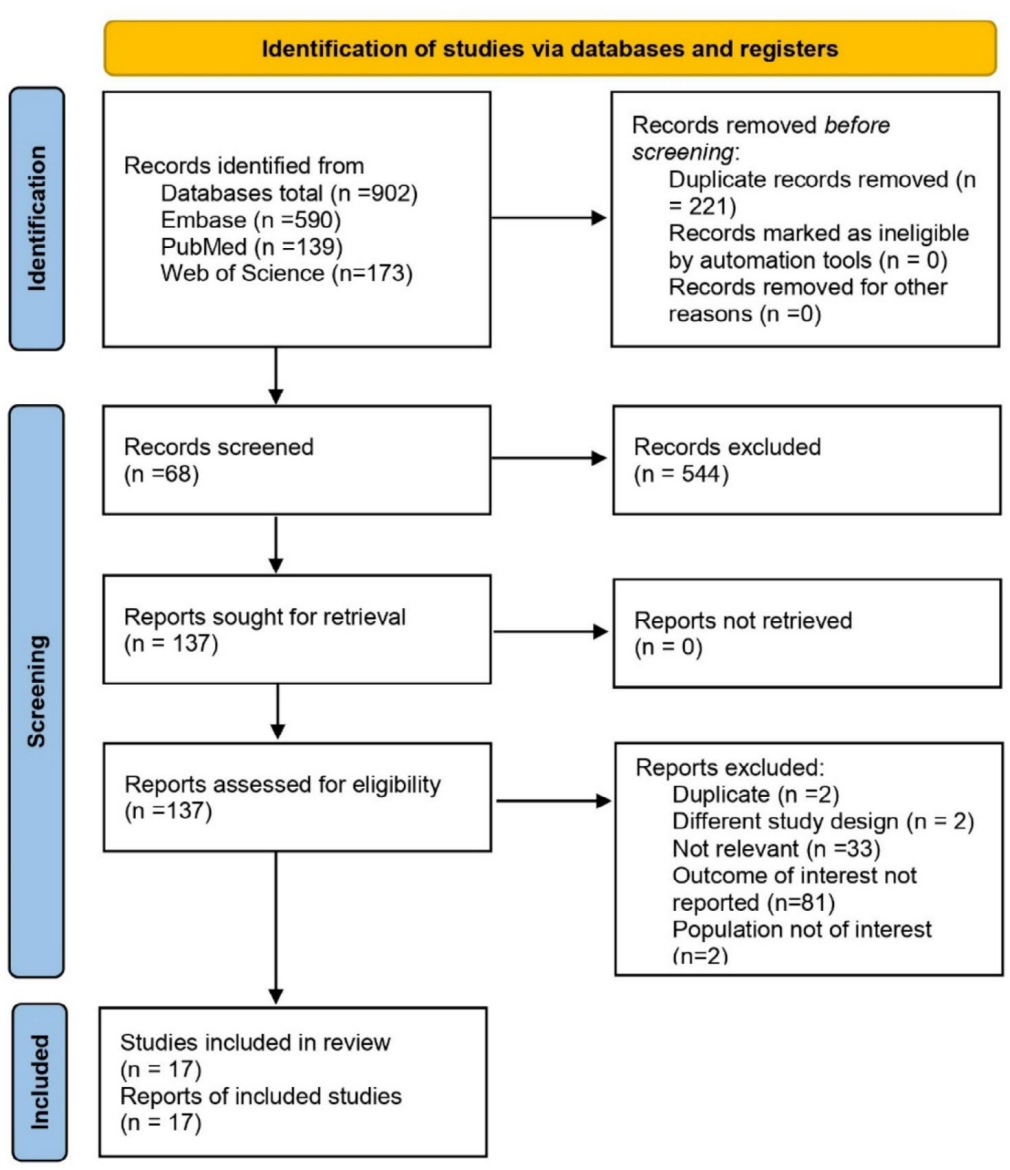
PRISMA flow diagram depicting article selection and screening process.

The literature search initially yielded 902 articles. Following the removal of 221 duplicate records, 681 records were retained for primary screening. At this stage, which involved screening titles and abstracts, 544 articles were excluded for various reasons, such as lacking a focus on the association between *H. pylori* infection and dyslipidemia, being irrelevant to the topic, or not containing original research data. Consequently, 137 articles advanced to the full‐text screening phase. Following a detailed assessment of these full texts, 17 studies [21, 26, 27, 28, 29, 30, 31, 32, 33, 34, 35, 36, 37, 38, 39, 40, 41] fulfilled the inclusion criteria and were incorporated into the final meta‐analysis. Figure 1 provides a flowchart of the study selection process.

### 3.2 Characteristics of Included Studies

The important characteristics of studies are presented in Table 1. The studies utilized different designs, including cross‐sectional, cohort, and case–control approaches. The research spanned a diverse range of settings, with studies conducted in countries such as China, South Korea, Ethiopia, the United States, Saudi Arabia, Cameroon, Italy, Bangladesh, and Israel. The study populations also varied, including patients undergoing routine health screenings, individuals suspected of *H. pylori* infection, and participants from different demographic backgrounds. The sample sizes across the studies ranged from smaller cohorts of 231 participants to large‐scale samples involving over 70 000 individuals. Various methods were employed to detect *H. pylori* , including stool antigen tests, antibody tests, and urea breath tests, while dyslipidemia was generally assessed based on established lipid profile criteria such as elevated cholesterol, LDL, triglycerides, and low HDL levels. Table S3 presents a detailed quality assessment of the studies.

**TABLE 1:** Characteristics of included studies.

Study	Study design	Country	Population	Total sample	Mean/median age	Male%	No of sample in *H. pylori* positive	No of sample in *H. pylori* negative	*H. pylori* detection method	Dyslipidemia criteria	OR/RR(95% CI) for dyslipidemia	Adjusted factors
Abdu 2020 [26]	Cross‐sectional study	Ethiopia	Individual patients suspected for *H. pylori* infection	369	41.03 ± 13.55	47.4%	173	196	Rapid antibody test strip Wondfo (one step *H. pylori* serum/plasma test)	NA	Dyslipidemia = OR = 2.628 (1.477–4.678)	Age, sex, BMI, smoking status, alcohol consumption, physical exercise, residence (urban vs. rural), occupational status, waist circumference
Izhari 2023 [27]	Case–control study	Saudi Arabia	*H. pylori* ‐infected patients and the *H. pylori* ‐negative cases	510	44.01 ± 13.58	NA	260	250	Stool antigen tests (SATs)	Cholesterol > 5.17 mmol/L, Triglycerides > 1.69 mmol/L, LDL‐C > 2.59 mmol/L, HDL‐C < 1 mmol/L (males) or < 1.3 mmol/L (females)	Hypercholesterolemia = 2.64 (1.824–3.848), hypertriglyceridemia = 3.24 (2.227–4.757), Increased LDL‐C levels = 2.174 (1.309–3.684), Decreased HDL‐C levels = 4.2 (2.937–6.321)	Age, gender, lipid profiles
Baeg 2016 [28]	Cross‐sectional study	South Korea	People who underwent routine health screening examinations	4030	54 (46–61)	*H. pylori* +ve = 60, *H. pylori* −ve = 56.9	1636	2027	C urea breath test	NA	NA	NA
Danny Nguefak Tali 2022 [29]	Cross‐sectional study	Cameroon	Dyspeptic subjects	363	47.53 ± 17.07	48.21%	239	124	During Esophagogastroduodenoscopy examinations (FOGD), biopsy samples were collected from the antrum, the fundus and the angulus for *H. pylori* detection	NA	High total choletrol = 2.5944 (1.5766–4.2692), high LDL‐C = 2.6794 (1.5839–4.5328), Low HDL‐C = 1.4103 (0.8588–2.3160), High TG = 1.1116 (0.6918–1.7861)	Sex, gender, income level, smoking, alcohol consumption, physical activity, history of hypertension, diabetes mellitus, obesity, medical history
Fang 2024 [30]	Case–control study	China	Patients with ACS	280	59.20 ± 12.98	Acs group = 81.43% Control group = 47.86%	NA	NA	Immunoblotting	NA	High total cholesterol = 1.37 (0.69–2.72), High triglyderides = 1.45 (0.71–2.98), Low HDL = 1.01 (0.53–1.91), High LDL = 0.94 (0.50–1.79)	Age, Sex, smoking status, hypertension, diabetes, lipid profiles
Haj 2021 [31]	Cross‐sectional study	Israel	Persons who performed the UBT between 2002 and 2012	12 207	54.4 ± 11.7	47.90%	6108	6099	NA	NA	NA	Age, sex, country of birth, residential socioeconomic status, smoking status, BMI, use of statins and diabetes medications
Hashim 2022 [32]	Cross‐sectional study	Ethiopia	Patients with symptoms of dyspepsia	346	33 ± 13	46.2%	174	172	Rapid antibody test strip	Abnormal lipid cut‐offs: TC > 200 mg/dL, TG > 150 mg/dL, LDL‐C > 130 mg/dL, HDL‐C < 40 mg/dL (NCEP guidelines).	Hypercholesterolemia = 0.555 (0.318–0.967)	Age, sex, blood pressure BMI, hip circumference (HC), alcohol consumption, and cigarette smoking
Kim 2016 [33]	Cross‐sectional study	South Korea	Healthy subjects	37 263	49.6	56.2%	21 968	15 278	*H. pylori* ‐specific immunoglobulin G antibody (IgG) test	NA	High LDL‐c:RR = 1.21 (1.12–1.30), Low HDL‐C: RR = 1.10 (1.01–1.18), High TG: RR = 1.03 (0.99–1.07)	Age, sex, education level, income level, smoking status, alcohol consumption, and physical inactivity
Nam 2015 [34]	Prospective cohort study	Korea	Participants who underwent routine checkup	4269	48.7 ± 8.6	*H. pylori* +ve = 60.9, *H. pylori* −ve = 58.3	2335	1935	Rapid urease test	NA	NA	Sex, BMI, smoking status, drinking status, education
Nigatie 2022 [35]	Cross‐sectional study	Ethiopia	*H. pylori* ‐infected patients attending an outpatient department	231	31 (IQR: 22–40)	*H. pylori* +ve = 45.3, *H. pylori* −ve = 53.5	117	114	NA	TC > 200 mg/dL, TG > 150 mg/dL, LDL‐C > 130 mg/dL, HDL‐C < 40 mg/dL (males) or < 50 mg/dL (females)	Dyslipidemia = 3.377 (1.637–6.966)	Age, sex, marital status, residence, education status, occupation Status, alcohol drinking habits, BMI, physical exercise, waist circumference (WC), hip circumference (HC)
Rahman 2021 [36]	Cross‐sectional study	Bangladesh	Adult subjects (≥ 18 years) of two villages of Bangladesh	1021	40.35 ± 15.56	*H. pylori* +ve = 38.8, *H. pylori* −ve = 31.5	418	349	NA	NA	NA	NA
Seo 2020 [37]	Retrospective study	South Korea	Adults who received health check‐ups	1065	45.2 (20–80)	67.50%	663	402	Rapid urease test (CLOtest, Delta West)	NA	Total cholesterol: Male = 1.007 (1.002–1.011), Female = 1.002 (0.973–1.031), Decreased HDL cholesterol: Male = 0.998 (0.978–1.019), Female 0.983 (0.968–0.998), LDL cholesterol: Male = 0.995 (0.985–1.004) Female = 0.999 (0.991–1.006), Triglyceride Male = 1.000 (0.998–1.003), Female = 0.997 (0.992–1.002)	Age, sex, metabolic syndrome
Tang 2019 [38]	Prospective cohort study	United States	Hispanic adults referred to the National Institutes of Health	270	47.6 ± 12.5	31.10%	89	181	Serology, stool antigen testing or histology via oesophagogastroduodenoscopy (OGD)	NA	Triglycerides = 1.01 (1.00–1.01), HDL = 0.95 (0.93–0.97), LDL = 1.01 (1.00–1.02)	Age, sex, and statin use
Wang 2022 [39]	Retrospective study	China	Participants who underwent physical examinations.	71 633	46.5 ± 12.5	*H. pylori* −ve = 57.6, *H. pylori* +ve = 58.6	24 745	46 888	C‐UBT	NA	NA	NA
Wawro 2019 [21]	Cohort study	Italy	*Helicobacter pylori* seropositivity in serum samples of the KORA study	2075	56.7 ± 13.4	50%	586	1489	NA	NA	Dyslipidemia: RR = 0.85 (0.62–1.14)	Age, sex, obesity, physical activity, education, alcohol intake, smoking, hypertension, gout, uric acid blood levels, and diabetes
Yang 2024 [40]	Retrospective study	China	Individuals who underwent health check‐ups at the Health Examination	60 535	49.8 ± 12.5	61.6%	22 416	38 119	C‐urea breath test	TC ≥ 240 mg/dL, TG > 200 mg/dL, HDL‐C < 40 mg/dL (men) or < 50 mg/dL (women), LDL‐C ≥ 130 mg/dL	Dyslipidemia = 1.14 (1.04–1.26)	Age, gender, hypertension, HbA1c, smoking, alcoholic consumption
Zhao 2019 [41]	Case–control study	China	*H. pylori* +ve and individuals *H. pylori* −ve	1982	41.2 ± 11.7	NA	617	617	NA	NA	NA	NA

Abbreviations: ACS, acute coronary syndrome; CI, confidence interval; C‐UBT, carbon urea breath test; FOGD, fibro‐optic gastroduodenoscopy; *H. pylori* , *Helicobacter pylori* ; HDL‐C, high‐density lipoprotein cholesterol; IQR, interquartile range; LDL‐C, low‐density lipoprotein cholesterol; NA, not available; NCEP, National Cholesterol Education Program; OGD, oesophagogastroduodenoscopy; OR, odds ratio; RR, relative risk; SAT, stool antigen test; TC, total cholesterol; TG, triglyceride.

### 3.3 Impact of *H. pylori* Infection on Total Cholesterol

**FIGURE 2:**
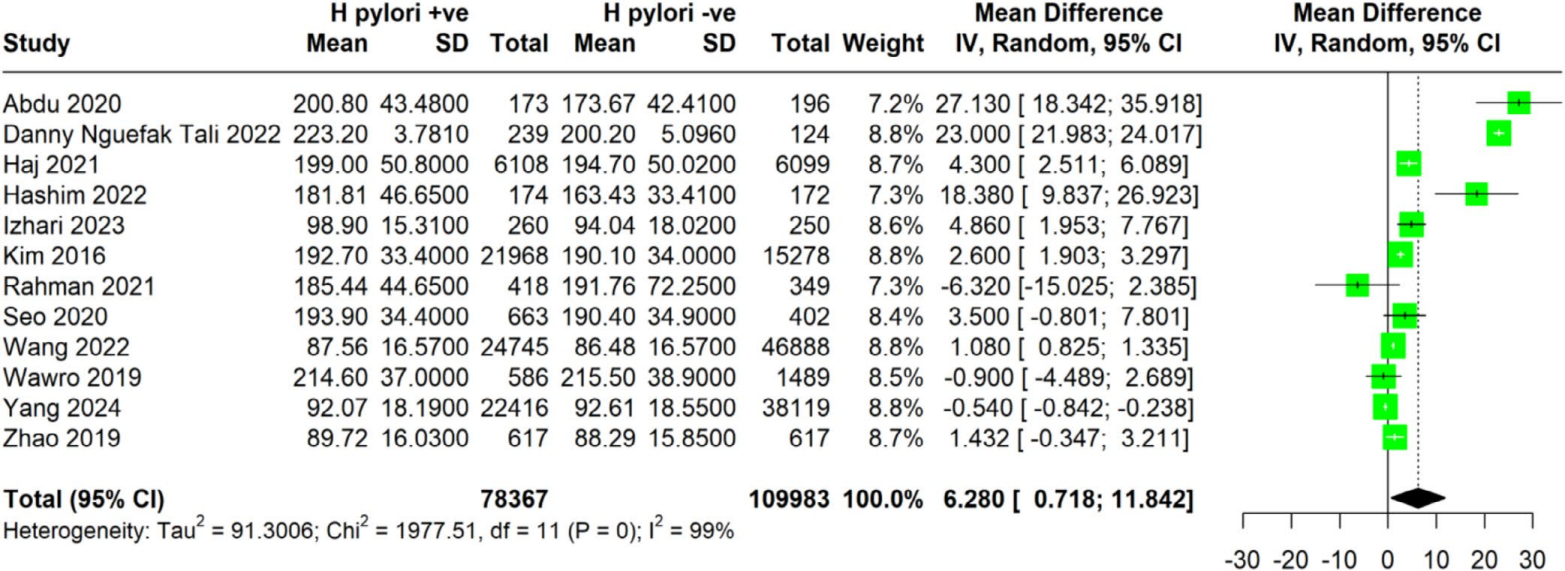
Meta‐analysis showing pooled mean difference of total cholesterol between H. pylori +ve and H. pylori −ve group.

The meta‐analysis compared the average cholesterol levels between individuals with and without *H. pylori* infection from multiple studies to determine the impact of *H. pylori* infection on total cholesterol levels (Figure 2). The results indicated that individuals with *H. pylori* infection had higher total cholesterol levels compared to those without the infection. The pooled MD was 6.28 mg/dL (95% CI: 0.718 to 11.842), showing a statistically significant increase in total cholesterol linked to *H. pylori* infection. The analysis included data from studies conducted across different populations, with a total of 78 367 *H. pylori* ‐positive and 109 983 *H. pylori* ‐negative participants. The heterogeneity was high (I^2^ = 99%), indicating significant variability between the studies. Despite the observed heterogeneity, the overall findings suggest a notable association between *H. pylori* infection and elevated total cholesterol levels.

**FIGURE 3:**
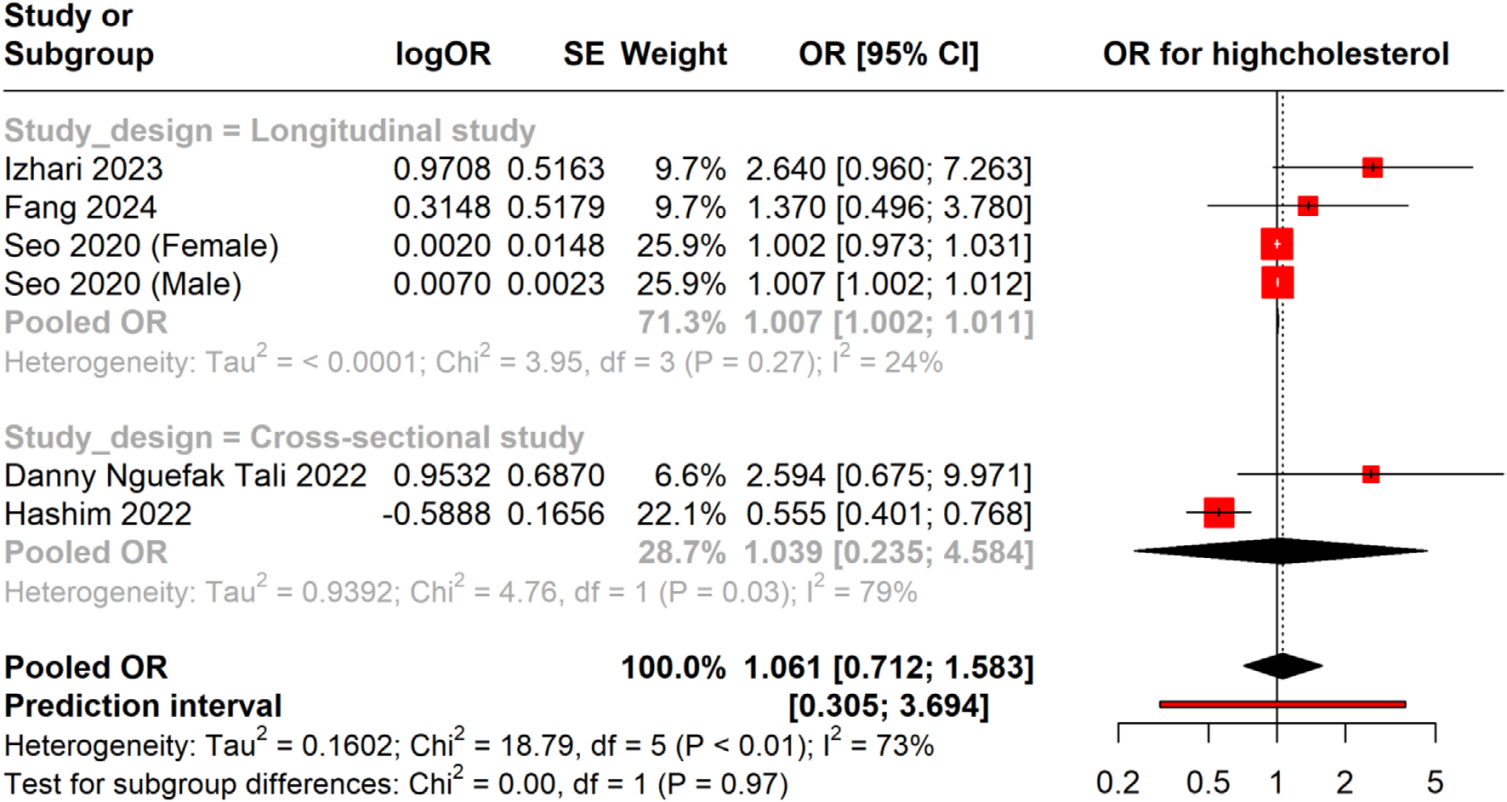
Meta‐analysis showing pooled OR of total cholesterol between *H. pylori* +ve and *H. pylori* −ve groups.

We also combined the adjusted OR for elevated LDL in patients infected with *H. pylori* from various study types, such as cross‐sectional and longitudinal designs. *H. pylori* infection and elevated risk of high LDL levels were not statistically significantly associated, as indicated by the total pooled OR of 1.044 (95% CI: 0.966 to 1.130). The prediction interval was 0.30 to 3.69. The risk of elevated cholesterol was not statistically significantly correlated with *H. pylori* infection, as indicated by the total pooled OR of 1.061 (95% CI: 0.712 to 1.583). With minimal heterogeneity, a subgroup analysis of four longitudinal studies revealed a pooled OR of 1.007 (95% CI: 1.002 to 1.011), suggesting a weak but statistically significant correlation between high cholesterol and *H. pylori* infection. There was no significant connection in the cross‐sectional studies subgroup, with a pooled OR of 1.039 (95% CI: 0.235 to 4.584). Nonetheless, this subgroup exhibited greater heterogeneity (I^2^ = 79%), indicating greater variation in the outcomes of the included research (Figure 3).

### 3.4 Impact of *H. pylori* Infection on Triglycerides

**FIGURE 4:**
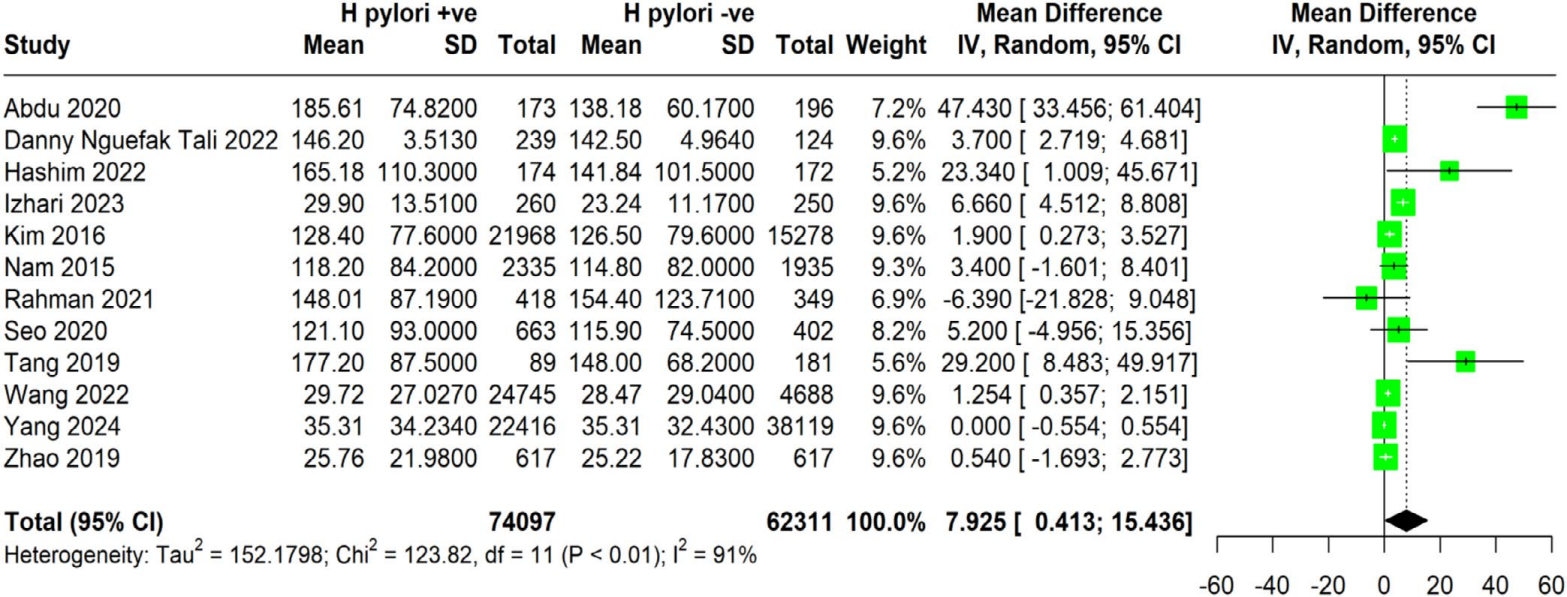
Meta‐analysis showing pooled mean difference of triglycerides between H. pylori +ve and H. pylori −ve group.

A meta‐analysis was conducted to assess the effect of *H. pylori* infection on triglyceride (TG) levels. By comparing the average TG levels between *H. pylori* ‐positive and *H. pylori* ‐negative individuals across multiple studies (Figure 4), the analysis found evidence of a statistically significant increase in TG levels associated with *H. pylori* infection. The pooled MD was 7.93 mg/dL (95% CI: 0.413 to 15.436). This analysis included a large sample size of 136,408 participants. However, significant heterogeneity (I^2^ = 91%) was observed among the studies, likely due to differences in study populations and methodologies. Despite this heterogeneity, the pooled MD suggests a modest elevation in TG levels in individuals with *H. pylori* infection.

**FIGURE 5:**
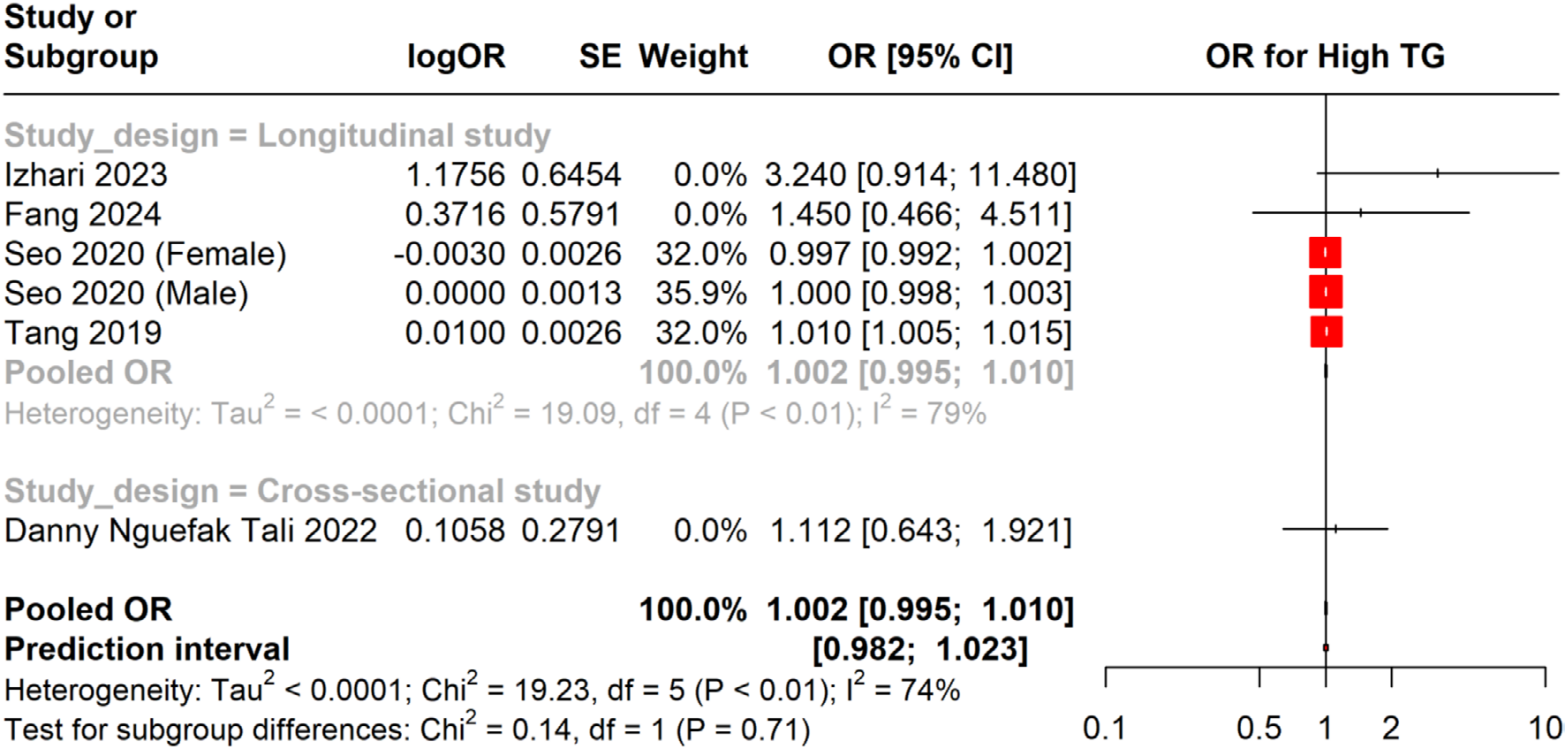
Meta‐analysis showing pooled OR of Triglycerides between *H. pylori* +ve and *H. pylori* −ve groups.

Additionally, we pooled the adjusted OR for high TG in *H. pylori* ‐infected individuals across different study designs, including longitudinal and cross‐sectional studies. The overall pooled OR was 1.002 (95% CI: 0.995 to 1.010), showing no statistically significant relation of *H. pylori* infection with the risk of high TG. The prediction interval was found to be 0.982 to 1.023. In the subgroup analysis for longitudinal studies, the pooled OR was 1.002 (95% CI: 0.995 to 1.010) with high heterogeneity (I^2^ = 74%). For cross‐sectional studies, the pooled OR was 1.112 (95% CI: 0.643 to 1.921), also showing no significant association. These results suggest no strong link between *H. pylori* infection and the risk of elevated TG levels, though the slight increase in MD warrants further exploration (Figure 5).

### 3.5 Impact of *H. pylori* Infection on LDL

**FIGURE 6:**
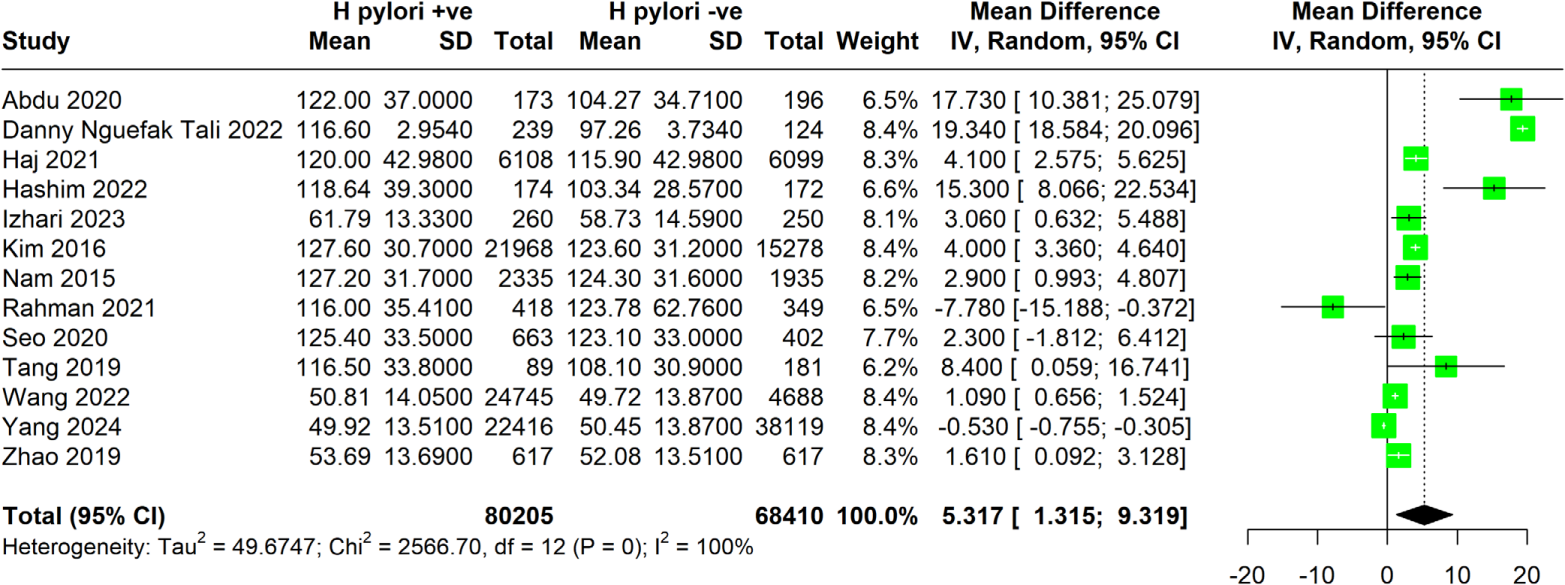
Meta‐analysis showing Pooled mean difference of LDL between H. pylori +ve and H. pylori −ve group.

The meta‐analysis evaluated the impact of *H. pylori* infection on low‐density lipoprotein levels by comparing the mean LDL values between the *H. pylori* ‐infected group and the *H. pylori* ‐uninfected group across various studies (Figure 6). The pooled MD was 5.32 mg/dL (95% CI: 1.315 to 9.319), indicating a statistically significant increase in LDL levels associated with *H. pylori* infection. The analysis involved 80 205 *H. pylori* ‐positive and 68 410 *H. pylori* ‐negative participants, totaling 148 615 individuals across multiple studies. High heterogeneity was observed (I^2^ = 100%), suggesting significant variability between studies. Despite this heterogeneity, the findings indicate a possible association between *H. pylori* infection and elevated LDL levels.

**FIGURE 7:**
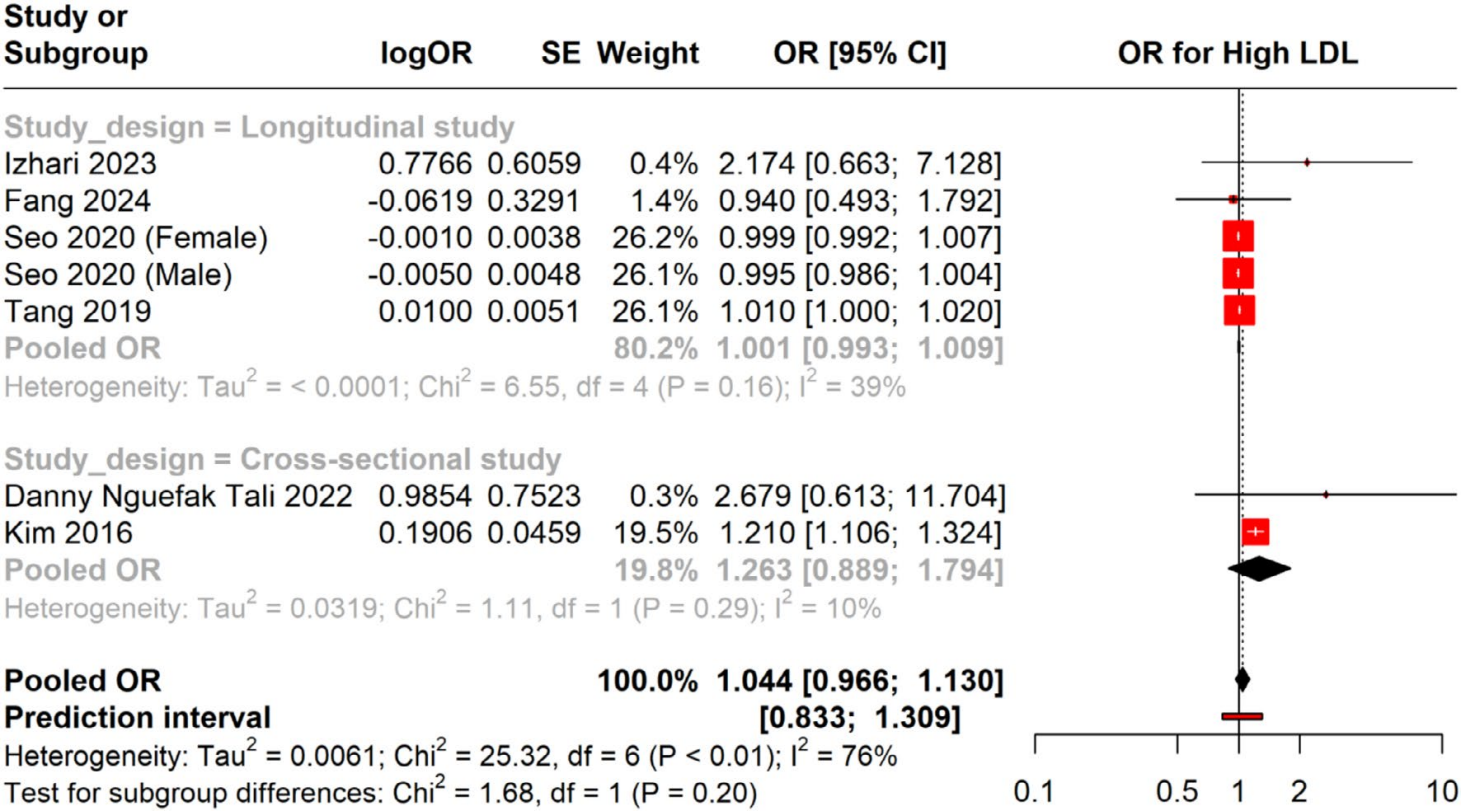
Meta‐analysis showing Pooled OR of LDL between H. pylori +ve and H. pylori −ve group.

Additionally, we pooled the adjusted OR for high LDL in individuals with *H. pylori* infection across different study designs, including longitudinal and cross‐sectional studies. The overall pooled OR was 1.044 (95% CI: 0.966 to 1.130), showing no statistically significant link between *H. pylori* infection and an increased risk of high LDL levels. The prediction interval was found to be 0.833 to 1.309. A subgroup analysis of longitudinal studies revealed an OR of 1.001 (95% CI: 0.993 to 1.009), showing a negligible association, suggesting no significant association with low heterogeneity (I^2^ = 39%). For cross‐sectional studies, the pooled OR was 1.263 (95% CI: 0.889 to 1.794), also showing no significant association but with low heterogeneity (I^2^ = 10%). The overall heterogeneity for the OR analysis was moderate (I^2^ = 76%), reflecting some variability among the included studies (Figure 7).

### 3.6 Impact of *H. pylori* Infection on HDL

**FIGURE 8:**
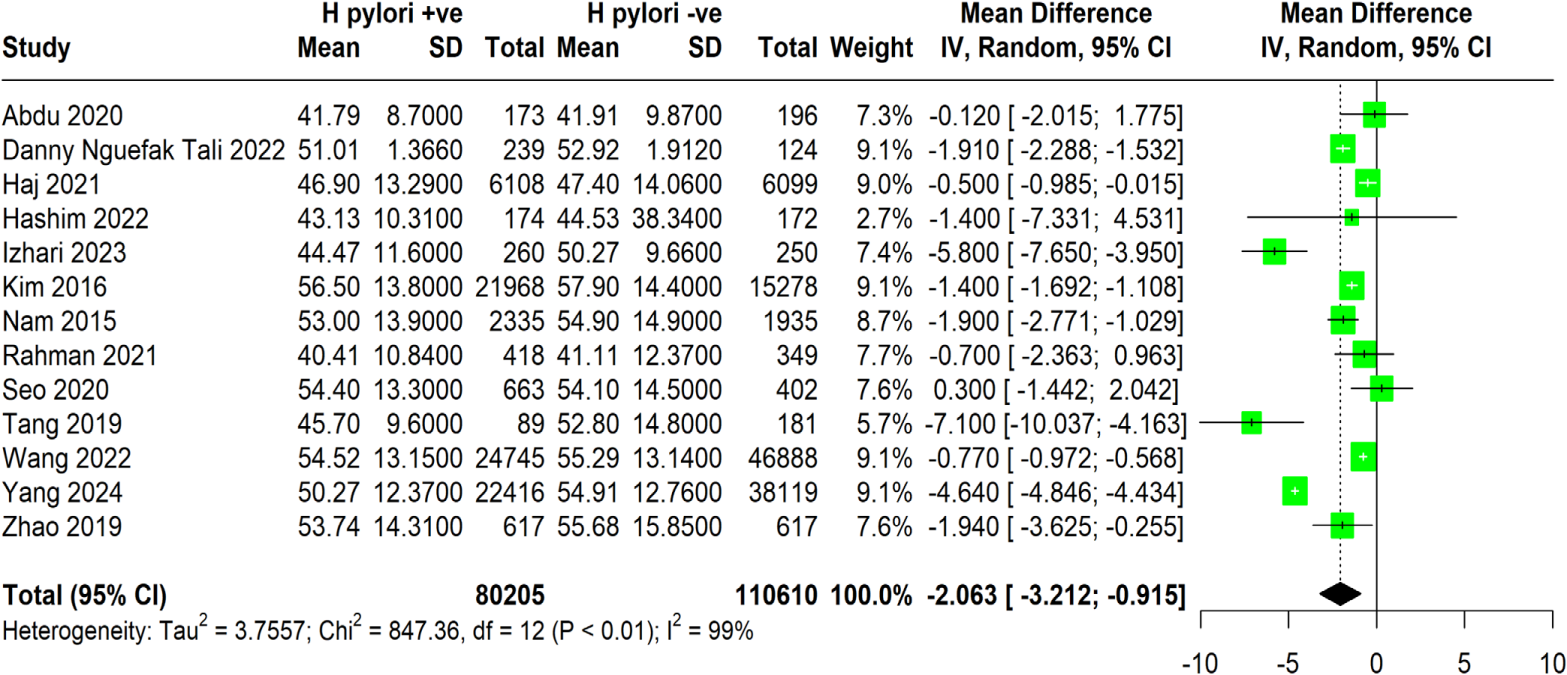
Meta‐analysis showing pooled mean difference of HDL between *H. pylori* +ve and *H. pylori* −ve groups.

The meta‐analysis assessed the effect of *H. pylori* infection on high‐density lipoprotein (HDL) levels by comparing the mean HDL values between *H. pylori* ‐positive and *H. pylori* ‐negative groups across various studies (Figure 8). The pooled MD was −2.06 mg/dL (95% CI: −3.212 to −0.915), indicating a statistically significant reduction in HDL levels associated with *H. pylori* infection. This analysis encompassed data from 80 205 participants who were *H. pylori* ‐positive and 110 610 who were *H. pylori* ‐negative across multiple studies. The heterogeneity was significant (I^2^ = 99%), indicating substantial variability among the studies. Despite this variability, the findings indicate a potential link between *H. pylori* infection and reduced HDL levels.

**FIGURE 9:**
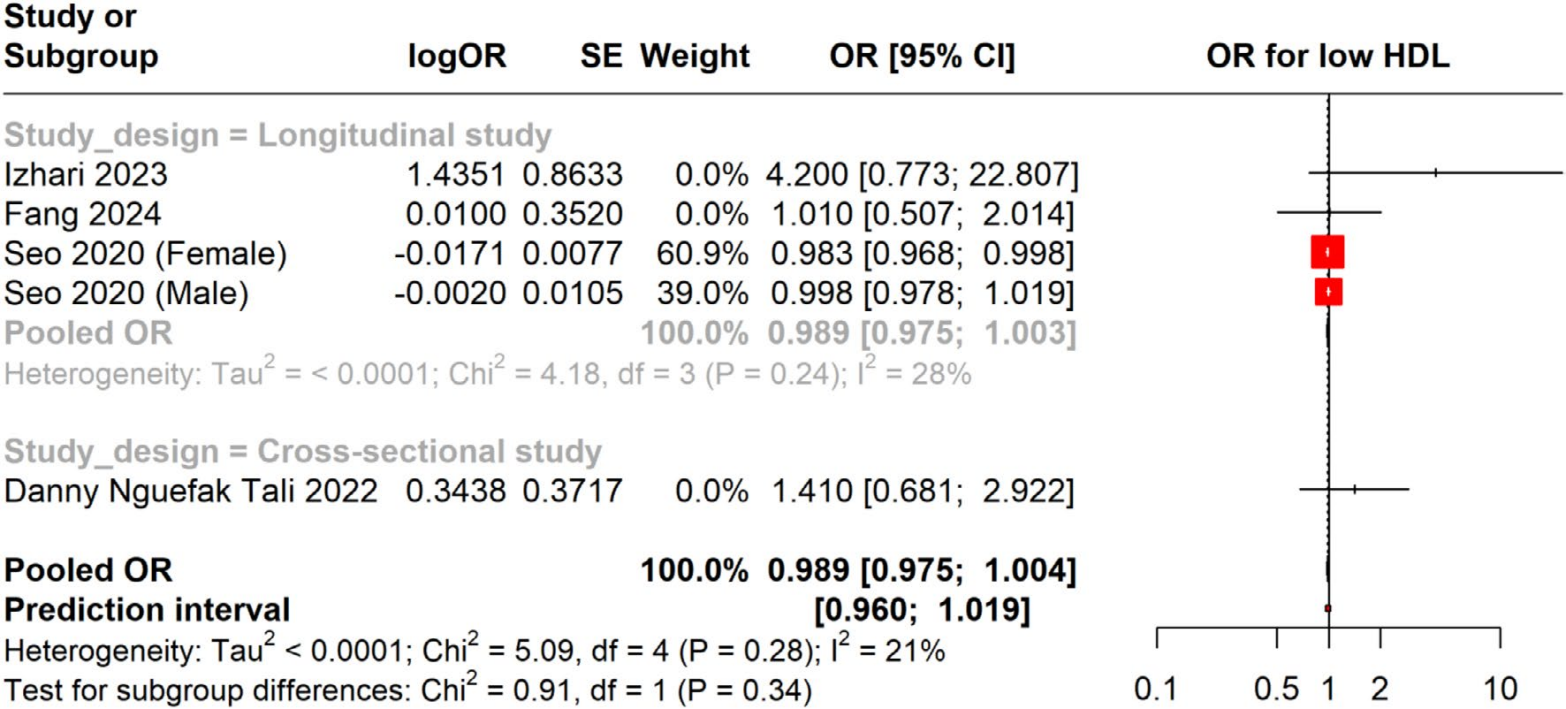
Meta‐analysis showing pooled OR of HDL between *H. pylori* +ve and *H. pylori* −ve groups.

Furthermore, we combined the adjusted OR for low HDL levels in individuals with *H. pylori* infection from various study designs, including both longitudinal and cross‐sectional studies. The overall pooled OR was 0.989 (95% CI: 0.975 to 1.004), revealing no statistically significant relation between *H. pylori* infection and the risk of low HDL levels. The prediction interval was found to be 0.960 to 1.019. In the subgroup analysis of longitudinal studies, the OR was 0.989 (95% CI: 0.975 to 1.003), with low heterogeneity (I^2^ = 28%), suggesting a consistent effect across these studies. For cross‐sectional studies, the OR was 1.410 (95% CI: 0.681 to 2.922), also indicating no significant association. The overall heterogeneity for the OR was low (I^2^ = 21%), reflecting limited variability among the studies in this analysis (Figure 9).

### 3.7 Publication Bias

Visual examination of the funnel plots and results from Egger's test reveal no significant publication bias for total cholesterol (Figure S1) (*p* = 0.241), LDL (Figure S2) (*p* = 0.233), and HDL (Figure S3) (*p* = 0.752), reinforcing the reliability of the pooled effect estimates. In contrast, the funnel plot for triglycerides (Figure S4) displays asymmetry, and Egger's test (*p* = 0.038) indicates a significant publication bias, which may be influenced by smaller studies reporting larger or more variable effects.

## 4 Discussion

This systematic review and meta‐analysis integrated available evidence regarding the association between *H. pylori* infection and dyslipidemia, drawing on data from 17 studies with over 150 000 participants. The results indicate that *H. pylori* infection is linked to elevated total cholesterol and LDL levels, alongside reduced HDL levels. However, the relationship between *H. pylori* infection and triglyceride levels, although significant in MD, did not translate into an increased risk when assessed through ORs.

Our meta‐analysis found that individuals with *H. pylori* infection had, on average, higher levels of total cholesterol and LDL, with a statistically significant MD observed across a large sample size. This result aligns with prior studies suggesting that chronic infections, such as *H. pylori* , may contribute to systemic inflammation, which in turn impacts cholesterol metabolism [42]. The observed elevation in total cholesterol and LDL among *H. pylori* ‐positive individuals might reflect the bacterium's role in enhancing systemic inflammation, potentially leading to hepatic upregulation of cholesterol synthesis pathways [43]. Elevated LDL levels are a well‐known risk factor for atherosclerosis, as they are prone to oxidative modification, which contributes to plaque formation in arterial walls [44]. Therefore, the association between *H. pylori* infection and dyslipidemia may have clinical relevance, particularly in populations at higher risk for cardiovascular diseases [33].

Our findings also revealed high heterogeneity (I^2^ = 99%) in cholesterol outcomes, indicating variability in study results. This heterogeneity could stem from differences in study populations, geographic variations in *H. pylori* strains, diagnostic criteria for infection, and variations in health status or diet, all of which can impact lipid profiles. For instance, the presence of CagA in *H. pylori* , which encodes a virulent toxin linked to stronger inflammatory responses, might partially explain variations in lipid outcomes across different populations [45]. Studies exploring strain‐specific effects could thus yield more granular insights into how different *H. pylori* strains influence lipid profiles and cardiovascular risk factors.

Interestingly, The analysis failed to demonstrate a statistically significant association between *H. pylori* infection and triglyceride levels, despite evidence from some studies indicating that chronic infections and inflammation can contribute to elevated triglycerides [46]. Elevated triglycerides are generally associated with conditions like metabolic syndrome and insulin resistance, and a mechanistic link between *H. pylori* infection and these pathways might be indirect or population‐dependent [47]. Furthermore, triglycerides are known to exhibit greater variability based on dietary intake, genetic predisposition, and physical activity, factors that may obscure any direct association with *H. pylori* [47]. In our subgroup analysis, studies focusing on longitudinal designs showed some correlation between *H. pylori* infection and slight increases in triglyceride levels, suggesting that the chronicity of infection might play a role in modulating triglycerides over time. However, due to high heterogeneity and potential publication bias detected for triglyceride data (Egger's test *p* = 0.038), caution is warranted in interpreting these findings, highlighting the need for more controlled studies to clarify the role of *H. pylori* in triglyceride metabolism. The findings regarding triglycerides also exhibit a discrepancy between the MD and ORs outcomes. The MD analysis identified a significant elevation in triglyceride levels among individuals with *H. pylori* infection, suggesting an acute effect of the infection on lipid metabolism. However, the OR analysis, which accounted for various confounding factors such as age, sex, comorbid conditions like diabetes and hypertension, and lifestyle choices including smoking and alcohol consumption, showed no significant association. These results suggest that the impact of *H. pylori* on triglycerides may be influenced by a wider array of factors, diluting the apparent direct effect seen in unadjusted analyses.

Our meta‐analysis found a small but statistically significant decrease in HDL levels among *H. pylori* ‐infected individuals, though no significant association was observed in pooled ORs for low HDL. Given that HDL plays a protective role against cardiovascular disease by facilitating reverse cholesterol transport, even a marginal reduction in HDL due to *H. pylori* infection could contribute to increased cardiovascular risk in affected individuals. Mechanistically, low‐grade systemic inflammation has been shown to reduce HDL concentrations, and *H. pylori* ‐associated inflammatory pathways might impact HDL synthesis or accelerate HDL degradation. Since HDL levels are also sensitive to lifestyle and dietary habits, future studies could consider adjusting for these variables to assess the independent impact of *H. pylori* on HDL more accurately. The MD analysis indicated a statistically significant reduction in HDL levels, suggesting an initial observation of lipid profile changes associated with *H. pylori* infection. Conversely, the OR analysis, which was adjusted for a comprehensive set of confounders including age, sex, diabetes, hypertension, statin use, smoking, alcohol consumption, BMI, and waist circumference, did not demonstrate a significant association. This divergence highlights the complex interplay of various factors influencing lipid metabolism in the context of *H. pylori* infection.

A previous systematic review explored the impact of eradication of *H. pylori* on circulating lipid levels [48]. This study combined evidence from both randomized controlled trials (RCTs) and non‐randomized controlled trials (non‐RCTs) to offer a thorough overview of how lipid profiles are altered after the eradication of *H. pylori* , a prevalent bacterial infection linked to various gastrointestinal disorders and possible cardiovascular consequences. It revealed that *H. pylori* eradication led to significant changes in certain lipid fractions. Specifically, there was a notable increase in HDL‐C levels. Additionally, TG levels also increased post‐eradication. However, the effect of *H. pylori* eradication on LDL‐C levels was minimal and statistically insignificant [48]. Its findings are comparable to our findings.

The observed associations between *Helicobacter pylori* infection and lipid abnormalities are likely mediated through complex, multifactorial mechanisms [49]. Chronic inflammation, one of the hallmark effects of *H. pylori* infection, induces a persistent inflammatory state in the gastric mucosa with systemic repercussions [50]. This is primarily facilitated through the release of pro‐inflammatory cytokines such as IL‐6 and TNF‐α, which may increase hepatic lipid synthesis and impair lipid clearance, thereby elevating cholesterol and LDL levels while potentially decreasing HDL concentrations [51]. Additionally, specific *H. pylori* strains express virulence factors like CagA, which provoke more intense inflammatory responses and are linked to severe metabolic disturbances, including lipid metabolism alterations [52]. Although our study did not directly assess the role of CagA, future analyses could benefit from exploring the association between CagA‐positive infections and dyslipidemia outcomes [52]. Furthermore, chronic *H. pylori* infection is associated with insulin resistance, a precursor to metabolic syndrome and an independent risk factor for dyslipidemia [53]. Insulin resistance may exacerbate dyslipidemia through mechanisms such as increased hepatic triglyceride synthesis and impaired lipid oxidation [54]. Emerging evidence also suggests that *H. pylori* infection can alter the composition of the gut microbiota, leading to shifts that may influence lipid metabolism [9]. These changes in gut microbial populations are known to affect lipid absorption, bile acid metabolism, and other critical aspects of host lipid regulation, presenting another potential link between *H. pylori* infection and dyslipidemia [9]. Investigating these pathways, particularly how *H. pylori* modifies the gut microbiome and impacts lipid profiles, could further elucidate the connections between infection and lipid dysregulation [46].

We observed statistically significant changes in lipid profiles associated with *Helicobacter pylori* infection. However, the clinical significance of these findings should be carefully considered. While the statistical analysis indicates changes in lipid levels, such as a slight increase in LDL and a decrease in HDL, the actual magnitude of these changes is relatively minor. This raises important questions about their clinical relevance. For instance, the observed differences in lipid levels, although statistically significant, may not necessarily translate into a meaningful impact on clinical outcomes such as cardiovascular risk or overall patient health. If *H. pylori* infection is confirmed to play a causal role in dyslipidemia, this might support *H. pylori* screening and eradication as a preventive measure for cardiovascular disease in certain high‐risk populations. While our meta‐analysis does not establish causation, it does highlight the need for clinicians to consider lipid profiling in *H. pylori* ‐infected patients, particularly those with other risk factors for dyslipidemia and cardiovascular disease. Additionally, these findings underscore the importance of dietary and lifestyle interventions for managing dyslipidemia in *H. pylori* ‐infected individuals, as these interventions might mitigate the adverse effects of infection on lipid metabolism.

The results of this meta‐analysis highlight the necessity for additional research to deepen our understanding of the relationship between *H. pylori* infection and lipid metabolism. Future investigations should prioritize longitudinal and interventional studies, such as prospective cohort studies and trials that examine lipid profile changes before and after *H. pylori* eradication therapy, to offer insights into causality. Additionally, research should delve into the inflammatory, metabolic, and microbiome‐related mechanisms by which *H. pylori* influences lipid metabolism, providing a more detailed understanding of the infection's systemic effects. Considering the geographical and strain‐specific differences in *H. pylori* virulence, it is also crucial to conduct population‐specific and strain‐specific analyses. Such targeted research would help clarify the observed heterogeneity in outcomes and strengthen the overall evidence base, facilitating more effective interventions and management strategies tailored to diverse populations.

While this study offers valuable insights, it is important to acknowledge several limitations. The analyses for total cholesterol, LDL, and HDL exhibited substantial heterogeneity, reflecting potential differences in population characteristics, study designs, diagnostic methods for assessing *H. pylori* infection, and lipid measurement protocols across the included studies. The high level of heterogeneity for some outcome variables limits the generalizability of the findings. Additionally, this study did not address the influence of specific *H. pylori* strains, such as CagA‐positive versus CagA‐negative, which are known to have differential effects on host inflammation and metabolism. We noted variations in the criteria used to define lipid abnormalities across different studies, with some using specific thresholds for LDL, HDL, and triglycerides. We addressed this by analyzing lipid levels as continuous variables, but recognize that these differences in definitions could impact the comparability and generalizability of our findings. Future research should focus on investigating these strain‐specific associations to provide a clearer understanding of the pathogenic and metabolic impacts of various *H. pylori* strains. Another limitation is the exclusion of non‐English language publications, which could introduce publication bias and overlook publications that could have potentially limited the comprehensiveness of the analysis, as studies conducted in non‐English‐speaking regions may not have been included.

## 5 Conclusion

Our study revealed a significant association between *H. pylori* infection and changes in lipid profiles, specifically increased total cholesterol and LDL levels, along with decreased HDL levels. These findings suggest that *H. pylori* infection may play a role in dyslipidemia through mechanisms related to the bacterium's inflammatory and metabolic effects. Given the considerable heterogeneity observed among the studies, further research is necessary to elucidate how *H. pylori* impacts lipid metabolism and to investigate the potential therapeutic benefits of eradicating *H. pylori* in the management of dyslipidemia. These results underscore the importance of recognizing chronic *H. pylori* infection as a potential contributor to lipid abnormalities and increased cardiovascular risk.

## Ethics Statement

The authors have nothing to report.

## Consent

The authors have nothing to report.

## Conflicts of Interest

The authors declare no conflicts of interest.

## Supporting information


**Data S1.** Supporting Information.

## Data Availability

The data are with the authors and available on request.
