# Supplementary material for: Association of Helicobacter pylori Infection and Risk of Dyslipidemia: A Systematic Review and Meta‐Analysis
Source: JGH Open. 2025 Mar 24;9(3):e70128. doi: 10.1002/jgh3.70128 (PMC11931453; doi:10.1002/jgh3.70128)
Supplement: Supplementary file 1 — Data S1. Supporting Information. [file JGH3-9-e70128-s001.docx]

**Supplementary Materials**

**Table S1.** PRISMA Checklist

**Table S2.** The adjusted search terms as per searched electronic databases

**Table S3.** Newcastle-Ottawa Scale for the quality assessment of studies

## **Table S1.** PRISMA Checklist

| **Section and Topic** | **Item #** | **Checklist item** | **Location where item is reported** |
| --- | --- | --- | --- |
| **TITLE** | | |  |
| Title | 1 | Identify the report as a systematic review. | 1 |
| **ABSTRACT** | | |  |
| Abstract | 2 | See the PRISMA 2020 for Abstracts checklist. (made as per the Journal guidelines) | 2 |
| **INTRODUCTION** | | |  |
| Rationale | 3 | Describe the rationale for the review in the context of existing knowledge. | 3 |
| Objectives | 4 | Provide an explicit statement of the objective(s) or question(s) the review addresses. | 3 |
| **METHODS** | | |  |
| Eligibility criteria | 5 | Specify the inclusion and exclusion criteria for the review and how studies were grouped for the syntheses. | 4 |
| Information sources | 6 | Specify all databases, registers, websites, organisations, reference lists and other sources searched or consulted to identify studies. Specify the date when each source was last searched or consulted. | 4, Table S3 |
| Search strategy | 7 | Present the full search strategies for all databases, registers and websites, including any filters and limits used. | Table S3 |
| Selection process | 8 | Specify the methods used to decide whether a study met the inclusion criteria of the review, including how many reviewers screened each record and each report retrieved, whether they worked independently, and if applicable, details of automation tools used in the process. | 4 |
| Data collection process | 9 | Specify the methods used to collect data from reports, including how many reviewers collected data from each report, whether they worked independently, any processes for obtaining or confirming data from study investigators, and if applicable, details of automation tools used in the process. | 4 |
| Data items | 10a | List and define all outcomes for which data were sought. Specify whether all results that were compatible with each outcome domain in each study were sought (e.g., for all measures, time points, analyses), and if not, the methods used to decide which results to collect. | 3 |
|  | 10b | List and define all other variables for which data were sought (e.g., participant and intervention characteristics, funding sources). Describe any assumptions made about any missing or unclear information. | 4, Table 1 |
| Study risk of bias assessment | 11 | Specify the methods used to assess risk of bias in the included studies, including details of the tool(s) used, how many reviewers assessed each study and whether they worked independently, and if applicable, details of automation tools used in the process. | Table S3 |
| Effect measures | 12 | Specify for each outcome the effect measure(s) (e.g. risk ratio, mean difference) used in the synthesis or presentation of results. | 5 |
| Synthesis methods | 13a | Describe the processes used to decide which studies were eligible for each synthesis (e.g. tabulating the study intervention characteristics and comparing against the planned groups for each synthesis (item #5)). | 4,5 |
|  | 13b | Describe any methods required to prepare the data for presentation or synthesis, such as handling of missing summary statistics, or data conversions. | NA |
|  | 13c | Describe any methods used to tabulate or visually display results of individual studies and syntheses. |  |
|  | 13d | Describe any methods used to synthesize results and provide a rationale for the choice(s). If meta-analysis was performed, describe the model(s), method(s) to identify the presence and extent of statistical heterogeneity, and software package(s) used. | 4.5 |
|  | 13e | Describe any methods used to explore possible causes of heterogeneity among study results (e.g. subgroup analysis, meta-regression). | 5 |
|  | 13f | Describe any sensitivity analyses conducted to assess robustness of the synthesized results. | 6 |
| Reporting bias assessment | 14 | Describe any methods used to assess risk of bias due to missing results in a synthesis (arising from reporting biases). | 5 |
| Certainty assessment | 15 | Describe any methods used to assess certainty (or confidence) in the body of evidence for an outcome. | NA |
| **RESULTS** | | |  |
| Study selection | 16a | Describe the results of the search and selection process, from the number of records identified in the search to the number of studies included in the review, ideally using a flow diagram. | Table S2 |
|  | 16b | Cite studies that might appear to meet the inclusion criteria, but which were excluded, and explain why they were excluded. | NA |
| Study characteristics | 17 | Cite each included study and present its characteristics. | 4,5 Table 1 |
| Risk of bias in studies | 18 | Present assessments of risk of bias for each included study. | Table S3 |
| Results of individual studies | 19 | For all outcomes, present, for each study: (a) summary statistics for each group (where appropriate) and (b) an effect estimate and its precision (e.g. confidence/credible interval), ideally using structured tables or plots. | Table 1 |
| Results of syntheses | 20a | For each synthesis, briefly summarise the characteristics and risk of bias among contributing studies. | 4 |
|  | 20b | Present results of all statistical syntheses conducted. If meta-analysis was done, present for each the summary estimate and its precision (e.g. confidence/credible interval) and measures of statistical heterogeneity. If comparing groups, describe the direction of the effect. | 5,4 Figure 2 |
|  | 20c | Present results of all investigations of possible causes of heterogeneity among study results. | 5, Figure 3 |
|  | 20d | Present results of all sensitivity analyses conducted to assess the robustness of the synthesized results. | Figure S2 |
| Reporting biases | 21 | Present assessments of risk of bias due to missing results (arising from reporting biases) for each synthesis assessed. | Figure S1-S4 |
| Certainty of evidence | 22 | Present assessments of certainty (or confidence) in the body of evidence for each outcome assessed. | NA |
| **DISCUSSION** | | |  |
| Discussion | 23a | Provide a general interpretation of the results in the context of other evidence. | 5,6, 7 |
|  | 23b | Discuss any limitations of the evidence included in the review. | 7 |
|  | 23c | Discuss any limitations of the review processes used. | 7 |
|  | 23d | Discuss implications of the results for practice, policy, and future research. | 7 |
| **OTHER INFORMATION** | | |  |
| Registration and protocol | 24a | Provide registration information for the review, including register name and registration number, or state that the review was not registered. | 3 |
|  | 24b | Indicate where the review protocol can be accessed, or state that a protocol was not prepared. | 3 |
|  | 24c | Describe and explain any amendments to information provided at registration or in the protocol. | NA |
| Support | 25 | Describe sources of financial or non-financial support for the review, and the role of the funders or sponsors in the review. | 8 |
| Competing interests | 26 | Declare any competing interests of review authors. | 7 |
| Availability of data, code and other materials | 27 | Report which of the following are publicly available and where they can be found: template data collection forms; data extracted from included studies; data used for all analyses; analytic code; any other materials used in the review. | Supplementary Materials |

**Table S2. The adjusted search terms as per searched electronic databases**

| Database | Search query | Results |
| --- | --- | --- |
| PubMed | **("helicobacter pylori"[MeSH Terms] OR ("helicobacter"[All Fields] AND "pylori"[All Fields]) OR "helicobacter pylori"[All Fields] OR "h pylori"[All Fields])**  **("dyslipidemia"[All Fields] OR "hyperlipidemia"[All Fields] OR "hypercholesterolaemia"[All Fields] OR "hypertriglyceridemia"[All Fields] OR "lipid disorder"[All Fields]) OR "dyslipid*"[All Fields] OR "dyslipidemia"[All Fields] OR "hyperlipid"[All Fields])** | 139 |
| EMBASE | **('helicobacter pylori' OR 'h pylori') AND ('dyslipidemia' OR 'hyperlipidemia' OR 'hypercholesterolaemia' OR 'hypertriglyceridemia' OR 'lipid disorder' OR 'dyslipid*' OR 'hyperlipid*')** | 590 |
| WOS  advanced | **("helicobacter pylori" OR "h pylori") AND ("dyslipidemia" OR "hyperlipidemia" OR "hypercholesterolaemia" OR "hypertriglyceridemia" OR "lipid disorder" OR "dyslipid*" OR "hyperlipid*") (All Fields)** | 173 |

**Table S3.** Quality assessment using Newcastle-Ottawa Scale

| **STUDY** | **SELECTION (max 4 points)** | | | | **COMPARABILITY (max 2 points)** | **OUTCOME (max 3 points)** | | | **SCORE (out of 6 for cross-sectional, 9 for cohort/case control)** |
| --- | --- | --- | --- | --- | --- | --- | --- | --- | --- |
|  | Representativeness | Selection | Ascertainment | Demonstration of the outcome of interest was not present at start of study | Comparability the basis of the design or analysis | Assessment of outcome | Was follow-up long enough for outcomes to occur? | Adequacy of the follow-up |  |
| Abdu 2020 (1) | 1 | 1 | 1 | NA | 1 | 1 | NA | NA | 5 |
| Izhari 2023 (2) | 1 | 1 | 1 | 1 | 1 | 1 | 1 | 1 | 8 |
| Baeg 2016 (3) | 1 | 1 | 1 | NA | 2 | 1 | NA | NA | 6 |
| Danny Nguefak Tali 2022 (4) | 1 | 1 | 1 | NA | 1 | 1 | NA | NA | 5 |
| Fang 2024 (5) | 1 | 1 | 1 | 1 | 2 | 1 | 1 | 1 | 9 |
| Haj 2021 (6) | 1 | 1 | 1 | NA | 1 | 1 | NA | NA | 5 |
| Hashim 2022 (7) | 0 | 1 | 1 | NA | 1 | 1 | NA | NA | 4 |
| Kim 2016 (8) | 1 | 1 | 1 | NA | 2 | 1 | NA | NA | 6 |
| Nam 2015 (9) | 1 | 1 | 1 | 1 | 1 | 1 | 1 | 1 | 8 |
| Nigatie 2022 (10) | 1 | 1 | 1 | NA | 1 | 1 | NA | NA | 5 |
| Rahman 2021 (11) | 1 | 1 | 1 | NA | 2 | 1 | NA | NA | 6 |
| Seo 2020 (12) | 1 | 1 | 1 | 1 | 1 | 1 | 1 | 1 | 8 |
| Tang 2019 (13) | 1 | 1 | 1 | 1 | 1 | 1 | 1 | 1 | 8 |
| Wang 2022 (14) | 1 | 1 | 1 | 1 | 1 | 1 | 1 | 1 | 8 |
| Wawro 2019 (15) | 1 | 1 | 1 | 0 | 2 | 1 | 1 | 1 | 8 |
| Yang 2024 (16) | 1 | 1 | 1 | 1 | 1 | 0 | 1 | 1 | 7 |
| Zhao 2019 (17) | 1 | 1 | 1 | 1 | 1 | 1 | 1 | 1 | 8 |


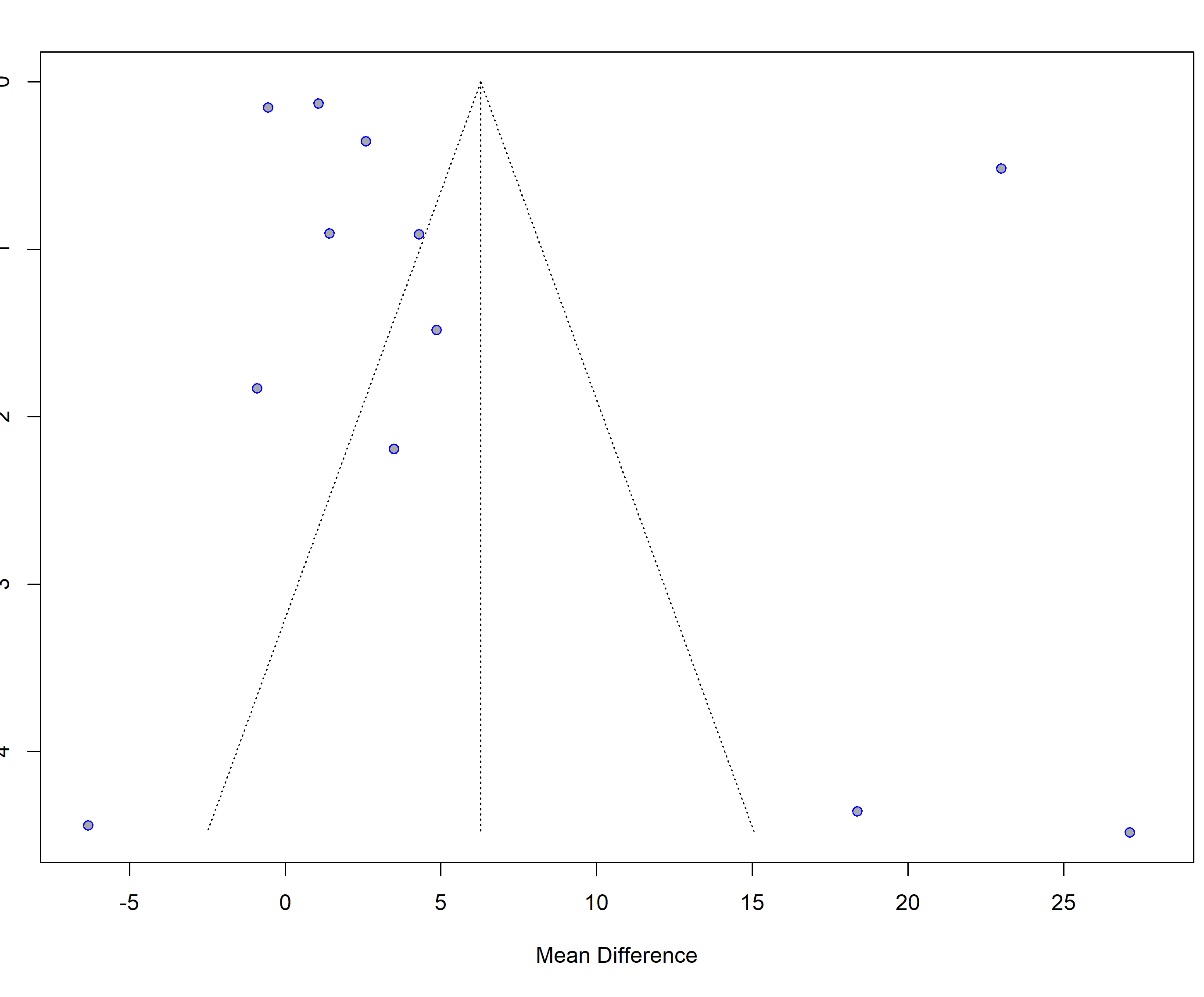


**Figure S1:** Funnel plot total cholesterol


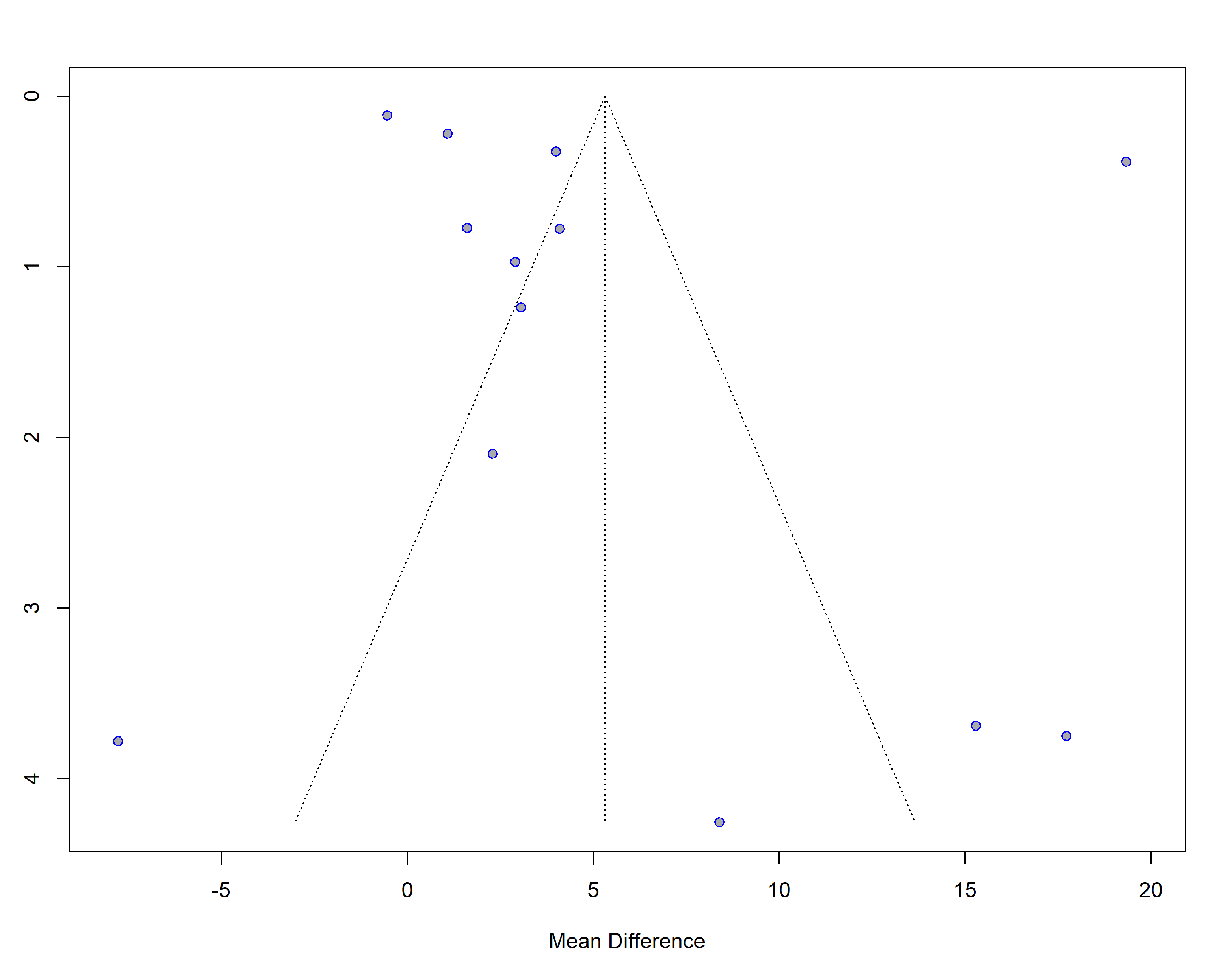


**Figure S2:** Funnel plot LDL


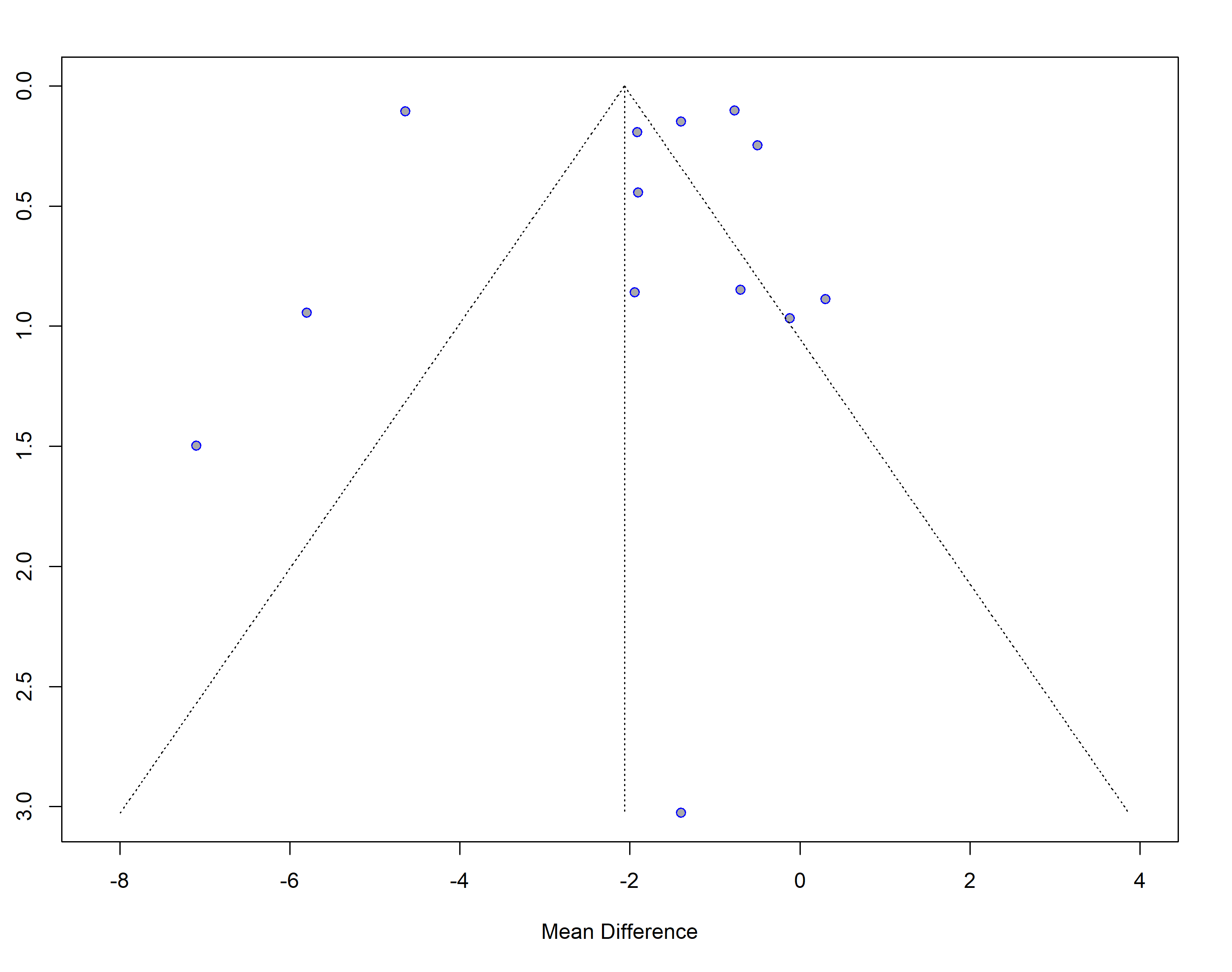


**Figure S3:** Funnel plot HDL


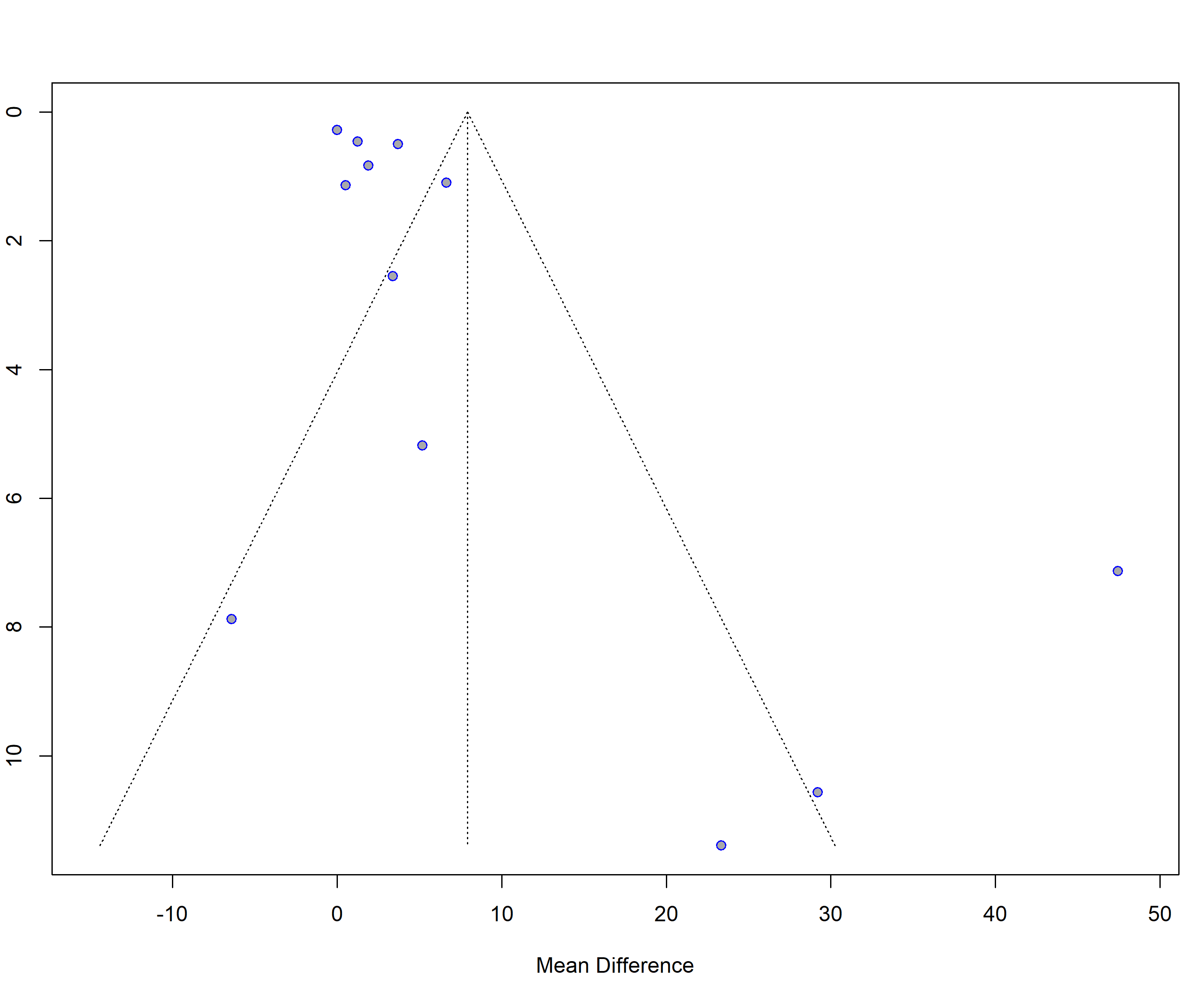


**Figure S4:** Funnel plot triglycerides

1. Abdu A, Cheneke W, Adem M, Belete R, Getachew A. Dyslipidemia and Associated Factors Among Patients Suspected to Have Helicobacter pylori Infection at Jimma University Medical Center, Jimma, Ethiopia. Int J Gen Med. 2020;13.

2. Asrar Izhari M, A. Al Mutawa O, Mahzari A, Ajmi Alotaibi E, A. Almashary M, Abdullah Alshahrani J, et al. Article Helicobacter pylori (H. pylori) Infection-Associated Dyslipidemia in the Asir Region of Saudi Arabia. Infection-Associated Dyslipidemia in the Asir Region of Saudi Arabia Life. 2023;13.

3. Baeg MK, Yoon SK, Ko SH, Noh YS, Lee IS, Choi MG. Helicobacter pylori infection is not associated with nonalcoholic fatty liver disease. World J Gastroenterol. 2016;22(8).

4. Danny Nguefak Tali L, Florice Nintewoue Faujo G, Laure Nguieguia Konang J, Paul Dzoyem J, Brigitte Mabeku Kouitcheu L. Relationship between active Helicobacter pylori infection and risk factors of cardiovascular diseases, a cross-sectional hospital-based study in a Sub-Saharan setting. Tali et al BMC Infectious Diseases. 2022;22.

5. Fang Y, Fan C, Li Y, Xie H. The influence of Helicobacter pylori infection on acute coronary syndrome and lipid metabolism in the Chinese ethnicity. Front Cell Infect Microbiol. 2024;14.

6. Haj S, Chodick G, Goren S, Na'amnih W, Shalev V, Muhsen K. Differences in glycated hemoglobin levels and cholesterol levels in individuals with diabetes according to Helicobacter pylori infection. Sci Rep. 2021;11(1).

7. Hashim M, Mohammed O, G/Egzeabeher T, Wolde M. The association of Helicobacter Pylori infection with dyslipidaemia and other atherogenic factors in dyspeptic patients at St. Paul's Hospital Millennium Medical College. Heliyon. 2022;8(5).

8. Kim TJ, Lee H, Kang M, Kim JE, Choi Y-H, Min YW, et al. Helicobacter pylori is associated with dyslipidemia but not with other risk factors of cardiovascular disease. Sci Rep. 2016;6.

9. Nam SY, Ryu KH, Park BJ, Park S. Effects of Helicobacter pylori infection and its eradication on lipid profiles and cardiovascular diseases. Helicobacter. 2015;20(2).

10. Nigatie M, Melak T, Asmelash D, Worede A. Dyslipidemia and Its Associated Factors Among Helicobacter pylori-Infected Patients Attending at University of Gondar Comprehensive Specialized Hospital, Gondar, North-West Ethiopia: A Comparative Cross-Sectional Study. J MultidiscipHealthc. 2022;15.

11. Rahman MM, Kibria MG, Sultana N, Akhter M, Begum H, Haque MA, et al. Seroprevalence of Helicobacter pylori and its association with metabolic syndrome in a rural community of Bangladesh. JGH Open. 2021;5(1).

12. Seo KI, Heo JJ, Kim SE, Park SJ, Park MI, Moon W, et al. Sex differences between <i>Helicobacter pylori</i> infection and cholesterol levels in an adult health checkup program. HELICOBACTER. 2020;25(4).

13. Tang DM, Chascsa DM, Chou JY, Ho N, Auh S, Wank SA, et al. Helicobacter pylori infection is strongly associated with metabolic syndrome, and weakly associated with non-alcoholic fatty liver disease, in a US Hispanic population. GastroHep. 2019;1(6).

14. Wang WJ, Fan MK, Gong R, Zhang YR, Zeng JC, Xu SP, Lin R. <i>Helicobacter pylori</i> infection is not an independent risk factor of non-alcoholic fatty liver disease in China. BMC GASTROENTEROLOGY. 2022;22(1).

15. Wawro N, Amann U, Butt J, Meisinger C, Akmatov MK, Pessler F, et al. <i>Helicobacter pylori</i> Seropositivity: Prevalence, Associations, and the Impact on Incident Metabolic Diseases/Risk Factors in the Population-Based KORA Study. FRONTIERS IN PUBLIC HEALTH. 2019;7.

16. Yang C, You N, Chen Y, Zhang J. Helicobacter pylori infection increases the risk of dyslipidemia in Chinese diabetic Population: a retrospective cross-sectional study. BMC Infect Dis. 2024;24(1).

17. Zhao MM, Krebs J, Cao X, Cui J, Chen DN, Li Y, et al. <i>Helicobacter pylori</i> infection as a risk factor for serum bilirubin change and less favourable lipid profiles: a hospital-based health examination survey. BMC INFECTIOUS DISEASES. 2019;19.
